# Contemporary Evidence on Interhospital Neonatal Transport as a Potentially Sepsis-Relevant Care Interval: A Scoping Review

**DOI:** 10.3390/medicina62091753

**Published:** 2026-09-11

**Authors:** Roni Octavian Damian, Lidia Boldeanu, Mihai Gabriel Cucu, Mirela Anişoara Siminel, Mihai Alexandru Butoi, Vlad Ionut Belghiru, Monica Iuliana Puticiu, Raluca Mihaela Tat, Bogdan Oprita, Mohamed-Zakaria Assani, Virginia Maria Radulescu, Cristiana Geormaneanu, Mihail Virgil Boldeanu, Luciana Teodora Rotaru

**Affiliations:** 1 Doctoral School, University of Medicine and Pharmacy of Craiova, 200349 Craiova, Romania; roni.damian@umfcv.ro (R.O.D.); mihai.butoi@umfcv.ro (M.A.B.); vlad.belghiru@umfcv.ro (V.I.B.);; 2 Department of Emergency Medicine and First Aid, University of Medicine and Pharmacy of Craiova, 200349 Craiova, Romania; cristiana.geormaneanu@umfcv.ro (C.G.); luciana.rotaru@umfcv.ro (L.T.R.); 3 Department of Microbiology, Faculty of Medicine, University of Medicine and Pharmacy of Craiova, 200349 Craiova, Romania; lidia.boldeanu@umfcv.ro; 4 Laboratory of Human Genomics, University of Medicine and Pharmacy of Craiova, 200638 Craiova, Romania; 5 Department of Neonatology, University of Medicine and Pharmacy of Craiova, 200349 Craiova, Romania; 6 Emergency Medicine Discipline, Faculty of Medicine, Vasile Goldis Western University of Arad, 310025 Arad, Romania; 7 Emergency Medicine Discipline, “Iuliu-Hațieganu” University of Medicine and Pharmacy, 400012 Cluj-Napoca, Romania; raluca.tat@umfcluj.ro; 8 Clinical Toxicology, Faculty of Medicine, “Carol Davila” University of Medicine and Pharmacy, 050474 Bucharest, Romania; 9 Department of Immunology, Faculty of Medicine, University of Medicine and Pharmacy of Craiova, 200349 Craiova, Romania; mihail.boldeanu@umfcv.ro; 10 Department of Medical Informatics and Biostatistics, Faculty of Medicine, University of Medicine and Pharmacy of Craiova, 200349 Craiova, Romania

**Keywords:** neonatal transport, neonatal sepsis, interhospital transfer, neonatal retrieval, early sepsis recognition, antimicrobial timing, evidence map, scoping review

## Abstract

*Background and Objectives*: Neonatal sepsis is time-sensitive, yet the role of interhospital transport in its early care pathway is poorly characterised. This scoping review systematically identified and classified contemporary neonatal transport variables, interventions, and organisational practices potentially relevant to early sepsis care, examined the evidentiary proximity of the available literature to neonatal sepsis, and identified priorities for direct investigation. *Materials and Methods*: PubMed/MEDLINE, Embase, Scopus, Web of Science, and the Cochrane Library were searched for English-language, peer-reviewed reports published from 1 January 2020 to 30 June 2026. Google Scholar, an academic search engine, and backward citation searching supplemented the database search. Two reviewers independently screened records; a third author charted data using a predefined matrix. Findings were reported according to PRISMA-ScR and synthesised descriptively because the mapping question and non-comparable concepts and outcomes did not support a pooled estimate. *Results*: Thirty-nine sources of evidence were included. They covered system organisation; respiratory and procedural stabilisation; haemodynamic, metabolic, and thermal management; medication and fluid use; adverse events; environmental exposures; and communication. Evidence was predominantly indirect: 24 sources were classified as indirect clinical, seven as indirect organisational, four as indirect technical, one as indirect experiential, and three as direct transport-process/indirect sepsis-outcome evidence. Few reports addressed infection-related processes, and none established that a transport intervention independently improved sepsis-specific outcomes. Antimicrobial timing, culture status, perfusion trajectories, and structured sepsis handover were rarely reported. *Conclusions*: Contemporary neonatal transport research identifies measurable care processes that may matter when sepsis is suspected, but the sepsis-specific effect of transport remains unproven. Prospective multicentre studies should directly link transport-phase processes with infection-defined populations and outcomes.

## 1. Introduction

Neonatal sepsis is a clinical syndrome of systemic illness caused by suspected or confirmed infection during the neonatal period; its early manifestations are often non-specific, yet deterioration towards organ dysfunction and shock can be rapid [1,2,3]. It remains an important cause of morbidity and mortality worldwide [1,2,3]. A global systematic review estimated an incidence of 2824 cases per 100,000 live births (95% confidence interval [CI], 1892–4194) and a case fatality of 17.6% (95% CI, 10.3–28.6), while emphasising substantial between-study heterogeneity and major geographic data gaps [4]. Despite advances in microbiological diagnosis, antimicrobial stewardship, and intensive care, deterioration may be rapid; emerging molecular diagnostic strategies may improve pathogen identification [5], but their value depends on timely recognition and coordinated care.

Interhospital neonatal transport may overlap with this early pathway. Critically ill neonates, including infants with suspected or evolving infection, may require transfer from facilities without advanced neonatal intensive care. During referral and transport, teams may reassess deterioration, stabilise respiratory or circulatory failure, preserve temperature and glucose, administer treatment, and communicate diagnostic uncertainty to the receiving unit. Specialist neonatal transport teams and dedicated services are therefore relevant to care continuity, although prior evidence does not establish a sepsis-specific effect [6,7].

The intersection between neonatal transport research and early sepsis care remains poorly defined. Existing transport studies and broader reviews principally address organisation, respiratory support, physiological stability, adverse events, environmental exposure, communication, or reporting variables rather than the evidentiary relationship of these domains to infection-defined care [7]. The specific knowledge gap is therefore not an absence of neonatal transport literature, but the absence of a sepsis-focused synthesis that distinguishes transport variables reported by included sources from evidence gaps and from variables proposed for future research. Consequently, the current literature is better suited to structured evidence mapping than to estimating the effect of a specific transport intervention.

This scoping review aimed to systematically identify, classify, and describe contemporary neonatal transport variables, interventions, and organisational practices; to assess how directly the available evidence addresses early sepsis care; and to delineate evidence gaps requiring prospective investigation in neonates with suspected or confirmed sepsis.

## 2. Materials and Methods

### 2.1. Review Design and Reporting Framework

This study was conducted as a scoping review because the question concerned the breadth, characteristics, and evidentiary relationship of transport research rather than the effectiveness of a single intervention. The review systematically identified, classified, and described sources across clinical, organisational, environmental, and communication domains and identified where sepsis-specific evidence was present or absent.

Reporting followed the PRISMA Extension for Scoping Reviews (PRISMA-ScR) [8]. We provide a completed PRISMA-ScR checklist in Supplementary File S1. We did not undertake a meta-analysis because the review synthesised different concepts and processes across non-comparable populations, exposures, and outcomes; this decision was not based on study design alone. Findings were charted and summarised descriptively across prespecified domains.

A working protocol and charting matrix were finalised before formal full-text data charting. Preliminary exploratory title/abstract mapping had already begun, and the protocol was not prospectively registered. The retained project record does not document changes to eligibility criteria, search concepts, or evidence-classification rules after the working protocol and matrix were finalised; however, it does not permit reliable reconstruction of whether wording or operational rules evolved during the preliminary mapping stage. We report this chronology explicitly and consider both the lack of prospective registration and the incomplete audit trail for the preliminary stage to be limitations.

### 2.2. Review Question and PCC Framework

The review question was as follows: Which contemporary interhospital neonatal transport variables, interventions, and organisational practices have been reported that may be relevant to early sepsis care, and where is direct sepsis-specific evidence lacking?

Eligibility was organised using a population–concept–context (PCC) framework. The population comprised neonates or separately identifiable neonatal subgroups. The concept comprised transport-phase clinical care, organisation, monitoring, adverse events, environmental exposure, communication, and outcomes that could plausibly intersect with suspected or evolving sepsis. The context was interhospital transport, neonatal retrieval, or mobile neonatal intensive care by ground or air. Throughout this review, interhospital neonatal transport is used as the umbrella term; interfacility transport and neonatal retrieval are retained when they reflect source terminology, service models, or search syntax.

Studies were not required to enrol only neonates with confirmed sepsis because preliminary mapping indicated that such evidence was sparse. Instead, each included source was classified by its relationship to sepsis evidence. This choice supports evidence mapping but does not permit indirect transport findings to be interpreted as sepsis-specific effects.

### 2.3. Eligibility Criteria

Peer-reviewed, English-language full-text reports were eligible when they included neonates or a separately identifiable neonatal subgroup; addressed interhospital neonatal transport, retrieval, or mobile neonatal intensive care; and reported at least one extractable clinical, organisational, environmental, communication, or outcome variable during the referral, stabilisation, or transport interval.

Eligible source types included observational cohorts, registry or database studies, cross-sectional studies, surveys, quality-improvement and implementation studies, service evaluations, and experimental or technical studies that directly measured the neonatal transport environment. The latter were retained to characterise environmental exposures, not to infer clinical effectiveness.

We excluded paediatric-only reports without separable neonatal data, intrahospital transport, incidental transport, reports without extractable transport-phase information, doctoral theses and other grey literature, narrative or systematic reviews and meta-analyses, guidelines, protocols, and individual case reports. Reviews and guidelines could inform background, citation chasing, or interpretation but were not counted among the 39 sources of evidence.

The search was restricted to 1 January 2020–30 June 2026 to focus on contemporary practice, including current transport equipment, telemedicine, advanced ventilation, environmental monitoring, and structured service models. Earlier landmark sources were used only for context. This pragmatic boundary may omit relevant earlier transport evidence and is acknowledged as a limitation rather than as a claim of historical comprehensiveness.

### 2.4. Information Sources and Search Strategy

PubMed/MEDLINE, Embase, Scopus, Web of Science, and the Cochrane Library were searched, with the last search conducted on 30 June 2026. Google Scholar was used as a supplementary academic search engine, not as a bibliographic database. Backward citation searching was performed after database screening using included reports and reviews that addressed neonatal interhospital transport, neonatal retrieval systems, transport safety, or transport-phase clinical and organisational processes.

Search concepts combined neonates with interhospital transport or retrieval and with sepsis-relevant care processes, including infection, antimicrobial or culture-related processes, respiratory and haemodynamic instability, thermal and metabolic management, adverse events, environmental exposure, communication, and handover. The retained database-specific search strings and eligibility clarifications are reported below and in Supplementary File S2. Query-level retrieval counts, database export files, source-specific identification logs, and Google Scholar ranking cut-offs were not available in the retained project files; consequently, the identification stage can be reported only through the reconciled aggregate counts. Re-running the searches after the original search date would interrogate updated databases and could not reproduce the historical source-specific yields; no retrospective counts were therefore generated.

The PubMed/MEDLINE strategy was

((“Infant, Newborn”[Mesh] OR neonat* OR newborn* OR preterm OR premature)

AND (“Transportation of Patients”[Mesh] OR “Patient Transfer”[Mesh] OR transport* OR transfer* OR retrieval OR “mobile intensive care” OR ambulance OR aeromedical OR interhospital) AND (sepsis OR infection OR “suspected sepsis” OR antibiotic* OR antimicrobial* OR culture* OR stabilization OR stabilisation OR respiratory OR ventilation OR hemodynamic OR haemodynamic OR perfusion OR glucose OR hypothermia OR temperature OR “adverse event*” OR handover OR communication OR noise OR vibration OR environmental)) AND (“2020/01/01”[Date—Publication]: “2026/06/30”[Date—Publication])

For Embase, the search was adapted as

(‘newborn’/exp OR neonat*:ti,ab OR newborn*:ti,ab OR premature:ti,ab OR preterm:ti,ab) AND (‘patient transport’/exp OR transport*:ti,ab OR retrieval:ti,ab OR interhospital:ti,ab OR ambulance:ti,ab OR aeromedical:ti,ab) AND (sepsis:ti,ab OR infection:ti,ab OR antibiotic*:ti,ab OR antimicrobial*:ti,ab OR culture*:ti,ab OR respiratory:ti,ab OR ventilation:ti,ab OR hemodynamic:ti,ab OR haemodynamic:ti,ab OR perfusion:ti,ab OR glucose:ti,ab OR temperature:ti,ab OR hypothermia:ti,ab OR “adverse event*”:ti,ab OR vibration:ti,ab OR noise:ti,ab OR handover:ti,ab OR communication:ti,ab) AND [2020–2026]/py

For Scopus, the following title–abstract–keyword strategy was used:

TITLE-ABS-KEY (neonat* OR newborn* OR preterm OR premature) AND TITLE-ABS-KEY(transport* OR transfer* OR retrieval OR interhospital OR ambulance OR aeromedical OR “mobile intensive care”) AND TITLE-ABS-KEY(sepsis OR infection OR antibiotic* OR antimicrobial* OR culture* OR respiratory OR ventilation OR hemodynamic OR haemodynamic OR perfusion OR glucose OR hypothermia OR temperature OR “adverse event*” OR vibration OR noise OR communication OR handover) AND PUBYEAR > 2019 AND PUBYEAR < 2027

For Web of Science, the topic search was

TS = (neonat* OR newborn* OR preterm OR premature) AND TS = (transport* OR transfer* OR retrieval OR interhospital OR ambulance OR aeromedical OR “mobile intensive care”) AND TS = (sepsis OR infection OR antibiotic* OR antimicrobial* OR culture* OR respiratory OR ventilation OR hemodynamic OR haemodynamic OR perfusion OR glucose OR temperature OR hypothermia OR “adverse event*” OR vibration OR noise OR communication OR handover)

Timespan: 2020–2026

For the Cochrane Library, the following search combinations were used: “neonatal transport”, “neonatal retrieval”, “interhospital neonatal transfer”, “neonatal transport AND sepsis”, “neonatal transport AND respiratory support”, “neonatal transport AND hypothermia”, and “neonatal transport AND adverse events”.

Google Scholar was used as a supplementary discovery source with targeted combinations of the following terms: “neonatal transport sepsis”, “interhospital neonatal transport”, “neonatal retrieval”, “neonatal transport respiratory support”, “neonatal transport adverse events”, “neonatal transport vibration”, “neonatal transport noise”, “neonatal transport handover”, “neonatal transport thermoregulation”, “neonatal transport antibiotics”, and “neonatal transport telemedicine”. The retained files did not preserve query-level result counts or ranking cut-offs for these searches; no source-specific Google Scholar count has therefore been reconstructed retrospectively.

### 2.5. Study Selection

After deduplication, two reviewers independently screened titles and abstracts against the prespecified criteria and independently assessed potentially eligible full texts. Disagreements were resolved through discussion and consensus; no automated screening tool was used.

Backward citation searching was undertaken after eligible full texts and relevant reviews had been identified. Supplementary records were deduplicated and subjected to the same eligibility and independent full-text assessment process as database records. Full-text exclusions were documented using one primary reason.

**Figure 1:**
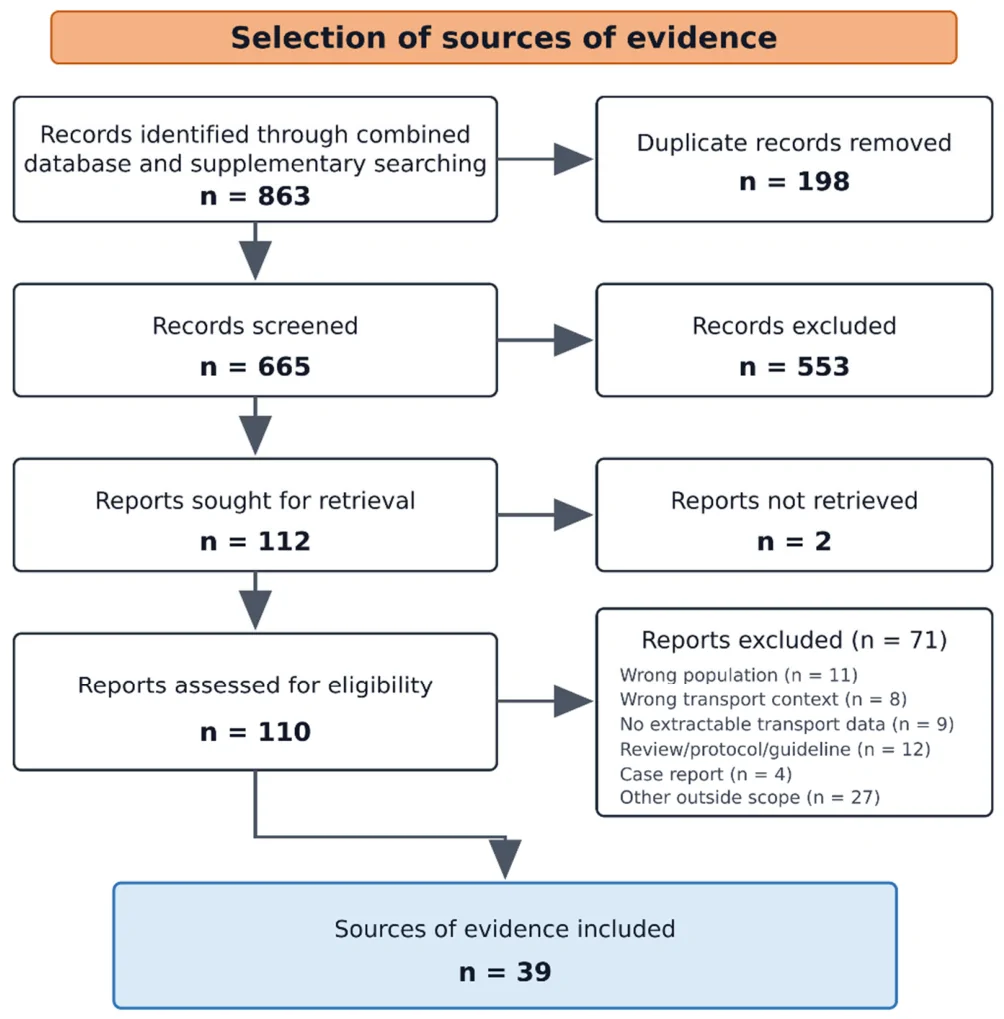
Preferred Reporting Items for Systematic Reviews and Meta-Analyses Extension for Scoping Reviews (PRISMA-ScR) flow diagram of source selection.

Records were classified as included sources of evidence, contextual or methodological references, or excluded reports. Only included sources contributed observations to the Results; contextual literature was reserved for the Introduction and Discussion. The selection process is presented in a PRISMA-ScR flow diagram (Figure 1).

### 2.6. Data Charting

A third author charted source-level data from the included sources using a predefined matrix [9,10]. Charting was not performed in duplicate. The retained source-level fields were citation, year, country or setting, design or source type, primary transport domain, relationship to sepsis evidence, reviewer-derived primary contribution to the evidence map, review decision, and source or DOI URL. Supplementary File S3 presents this source-classification and summary matrix and does not purport to contain a complete source-level extraction of sample sizes, outcome definitions, or study-specific clinical results.

Because source-level charting was completed by one author, transcription or interpretive errors cannot be excluded; this is reported as a review limitation. No information absent from the retained source-level records was imputed, and fields that were not preserved in a completed form were not reconstructed retrospectively.

### 2.7. Data Items and Synthesis

Charted items were organised into eight domains: system organisation and transport-team performance; medication, fluids, and infection-related documentation; respiratory and procedural stabilisation; haemodynamic, metabolic, and thermal stabilisation; adverse events and physiological deterioration; environmental exposures; communication, handover, telemedicine, and family experience; and post-transport outcomes. These domains were initially derived deductively from the PCC concept and predefined charting fields and were then refined descriptively during synthesis to group the variables represented in the included sources.

Each source was assigned to one primary transport domain according to its dominant transport-phase focus; where a source addressed several domains, secondary contributions could inform the narrative synthesis but were not multiply counted in domain-level classification. The synthesis reports the number and characteristics of sources and describes which variables were measured. The core-variable summaries were derived from the source-level matrix, whereas the evidence-gap column represents the review authors’ synthesis of absent, sparse, or inconsistently reported items. Results were not pooled because no sufficiently comparable, outcome-specific body of evidence was identified for an effect, prevalence, or diagnostic-accuracy estimate. Interpretation of potential clinical relevance was reserved for the Discussion.

No formal assessment of publication bias was performed because there was no pooled effect estimate or sufficiently homogeneous body of studies. Possible publication, language, indexing, and selective-reporting biases were considered when interpreting the evidence map.

### 2.8. Methodological Characterisation

The retained methodological characterisation comprised study design or source type, setting, primary transport domain, and relationship to sepsis evidence. Source-reported clinical variables informed the narrative interpretation where documented, but a completed source-level extraction of sample sizes, outcome definitions, and study-specific limitations was not retained for supplementary reporting. No formal critical appraisal was incorporated into source selection, weighting, or synthesis.

Accordingly, the evidence-relationship categories describe evidentiary proximity only and should not be interpreted as methodological-quality or certainty ratings.

### 2.9. Relationship to Sepsis Evidence and Interpretive Boundaries

Included sources were classified as direct transport-process/indirect sepsis-outcome evidence when transport processes, infection-relevant diagnoses, treatment processes, or transport conditions were reported with clinical outcomes that were not sepsis-specific; indirect clinical evidence when transport-phase clinical variables were reported in mixed neonatal populations without an explicit sepsis-specific process–outcome link; indirect organisational evidence when the dominant contribution concerned service configuration, workflow, performance, or implementation; indirect experiential evidence when the dominant contribution concerned parent or family perspectives; and indirect technical evidence when the dominant contribution concerned environmental or equipment exposures without a clinical sepsis outcome. Each source received one primary evidence category, selected according to the contribution most closely aligned with the review question; secondary features were retained in the narrative interpretation but were not multiply classified.

These labels describe evidentiary proximity, not quality or certainty. We did not use low, moderate, or high evidence categories and did not apply GRADE because no outcome-specific, comparable body of effect estimates was available. Contextual references did not contribute observations to the Results.

## 3. Results

### 3.1. Source Selection

The retained aggregate selection record reported 863 records; 198 duplicates were removed and 665 records underwent independent title/abstract screening. Of 112 reports sought for retrieval, 2 were not retrieved and 110 were assessed in full text. Seventy-one reports were excluded: wrong population (*n* = 11), wrong transport context or incidental/intrahospital transport (*n* = 8), no extractable transport-phase variables (*n* = 9), review/protocol/guideline design (*n* = 12), case report design (*n* = 4), and technical, illustrative, or contextual focus outside the eligibility definition (*n* = 27).

Thirty-nine sources of evidence were included. Reviews, guidelines, case reports, protocols, and other contextual references were not counted and did not contribute observations to the Results.

### 3.2. General Characteristics of the Included Sources

The 39 included sources covered regional retrieval systems, national networks, low-resource referral pathways, ground and air transport, and specialised neonatal emergency services [11,12,13,14,15,16,17,18,19,20,21,22,23,24,25,26,27,28,29,30,31,32,33,34,35,36,37,38,39,40,41,42,43,44,45,46,47,48,49]. Designs included observational cohorts, registry analyses, surveys, quality-improvement or implementation studies, service evaluations, and direct measurements of transport environmental exposure.

The corpus was not organised around a common intervention or outcome. Sources measured different transport processes and populations, and infection-defined subgroups were uncommon. No comparable body of prevalence estimates or intervention effects was identified; accordingly, the findings were organised descriptively rather than pooled.

Table 1 reports source characteristics and each source’s relationship to sepsis evidence. Contextual and methodological references are discussed separately and are not presented in Table 1.

### 3.3. Evidence Domains and Sepsis-Relevance Classification

Evidence was predominantly indirect. Of the 39 included sources, 24 were classified as indirect clinical, seven as indirect organisational, four as indirect technical, one as indirect experiential, and three as direct transport-process/indirect sepsis-outcome evidence. Most sources described processes that could matter during deterioration—such as respiratory escalation, temperature instability, perfusion-related scores, medication delivery, adverse events, environmental exposure, mobilisation, or handover—but did not define suspected or confirmed sepsis as the population or outcome.

A small subset reported infection-related diagnoses or medication processes, yet reporting of culture status, antimicrobial timing, and infection-specific outcomes was insufficient for direct effect inference. The classification in Table 1 therefore indicates proximity to the sepsis question and does not represent direct evidence of sepsis effects, a methodological-quality grade, or a certainty rating.

Table 2 summarises the extent and character of reporting across the eight domains. The transport-variable column contains variables represented in the included sources, whereas the evidence-gap column is a reviewer-derived interpretation of missing, sparse, or inconsistently reported information. Variables proposed for future prospective research are presented separately in Section 3.11 and are not represented as observed findings. The table does not assign certainty levels; its purpose is descriptive rather than evaluative.

### 3.4. System Organisation and Transport-Team Performance

Organisation and service performance were reported in service evaluations, observational cohorts, surveys, and quality-improvement studies [11,12,14,16,17,22,24,27,31,34,43,44,45]. Reported variables included team or service configuration, procedural and stabilisation time, referral conditions, mobilisation, transport mode, and operational outcomes.

The studies used different service models and outcome definitions. None directly tested whether a shorter mobilisation interval or a particular organisational model improved a sepsis-specific outcome.

### 3.5. Treatment Timing, Medication Use, and Culture-Related Documentation

One included study specifically described medications and intravenous fluids administered during neonatal transport [29]. Several referral and outcome studies recorded diagnoses, treatment, or admission status, but infection-related variables were not consistently isolated [15,17,22,43].

Across the included corpus, time of first sepsis concern, blood-culture status, and antibiotic name, dose, and time were not reported as a standardised transport timeline. Consequently, no included study supported an estimate of the effect of transport-phase antimicrobial administration on sepsis outcomes.

### 3.6. Respiratory Support and Procedural Stabilisation

Respiratory support was reported in observational studies, service evaluations, and surveys [19,25,35,37,39,41,46,48]. The reported interventions and variables included conventional and high-frequency ventilation, CPAP or other non-invasive support, equipment availability, escalation, and respiratory interventions during ground or aeromedical transfer.

Advanced support was feasible in the reported service contexts, but populations, indications, devices, and outcomes differed. Infection-defined respiratory subgroups and sepsis-specific outcomes were generally not reported.

### 3.7. Haemodynamic, Metabolic, and Thermal Stabilisation

Physiological scoring and admission-condition studies reported temperature, oxygenation, perfusion-related variables, glucose, or composite transport scores [20,21,22,23]. Temperature-targeted transport interventions were also reported, predominantly for hypoxic–ischaemic encephalopathy rather than infection [26,30,33,36,38].

Repeated perfusion and glucose trajectories were less consistently described than respiratory support or temperature. The included studies did not validate any transport score or thermal intervention specifically for neonatal sepsis.

### 3.8. Adverse Events and Physiological Deterioration

Adverse events, unintended events, vital-sign trajectories, or transport-related risk factors were reported in several observational sources [21,27,42,43,47]. Event types included physiological deterioration, equipment or procedural problems, and unplanned escalation, although definitions and ascertainment varied.

Most sources did not link events with infection suspicion, culture or antimicrobial status, or a sepsis-defined outcome. The evidence therefore describes transport safety reporting rather than the effect of transport on sepsis progression.

### 3.9. Environmental Exposures and Transport-Related Stress

Five included sources measured or described sound, vibration, air-transport conditions, or related environmental exposures [18,32,40,45,49]. These comprised technical or exposure-monitoring studies and a population-based air-transport cohort.

The sources showed that transport exposures can be quantified, but they did not establish sepsis-specific exposure thresholds or demonstrate that exposure reduction improves clinical outcomes.

### 3.10. Communication, Handover, Telemedicine, and Family-Centred Care

Telemedicine implementation and parental needs were examined in two included studies [13,28]. Referral conditions and organisational studies also reported aspects of communication or transfer coordination [22,24].

No included source evaluated a standardised infection-specific handover dataset. Maternal infection risks, time of sepsis concern, culture status, and antimicrobial timing were not consistently reported together.

### 3.11. Summary of Evidence Gaps and Implications for a Transport-Sepsis Dataset

Across domains, the most recurrently reported variables concerned respiratory support, temperature, service organisation, transport scores, adverse events, and environmental exposure. The least consistently reported sepsis-specific items were the time of first infection concern, culture status, antimicrobial timing, repeated perfusion and glucose measures, and structured handover.

Because populations and outcomes were not infection-defined, the evidence map cannot determine whether transport caused benefit or harm in neonatal sepsis. It identifies measurable candidate processes for future prospective study.

Separately from the variables reported by the included sources and the evidence gaps identified in Table 2, the review authors propose that a future minimum dataset could prospectively capture referral time, infection suspicion, maternal and perinatal risk factors, culture and antimicrobial timelines, respiratory and perfusion trajectories, temperature and glucose, fluids or vasoactive support, adverse events, handover completion, admission condition, organ support, length of stay, and mortality. This proposed dataset is hypothesis-generating, has not been validated, and should not be interpreted as an observed effect or an evidence-based minimum standard.

In summary, the 39 included sources describe a clinically active transport interval, but direct sepsis-specific evidence is sparse. The principal contribution of the Results is to delineate what has and has not been measured.

## 4. Discussion

### 4.1. Principal Findings

This scoping review examined 39 contemporary sources showing that interhospital neonatal transport commonly involves active clinical care, service organisation, physiological monitoring, environmental exposure, and handover [11,12,13,14,15,16,17,18,19,20,21,22,23,24,25,26,27,28,29,30,31,32,33,34,35,36,37,38,39,40,41,42,43,44,45,46,47,48,49]. The map also shows a clear evidentiary gap: most sources were not designed around suspected or confirmed sepsis and did not report culture or antimicrobial timelines.

The most defensible interpretation is therefore that transport contains processes potentially relevant to early sepsis care, not that transport or a specific transport intervention improves sepsis outcomes. This distinction is maintained throughout the revised manuscript.

Respiratory escalation, medication and fluid delivery, temperature and glucose status, adverse events, environmental exposures, mobilisation, referral, and handover are measurable candidate variables. Their relevance to sepsis is not equivalent: infection suspicion, culture status, antimicrobial timing, perfusion, and infection-specific handover have a comparatively direct conceptual relationship to early sepsis care, whereas noise, vibration, transport mode, and general adverse-event reporting have a more indirect or contextual relationship. Across both groups, inconsistent definitions and weak linkage to infection-defined outcomes prevent effectiveness conclusions.

Contextual reviews and reporting frameworks support this interpretation by identifying heterogeneity in neonatal transport safety metrics, national datasets, reported variables, environmental stressors, communication, and family support [50,51,52,53,54,55,56]. These contextual sources were not included in the 39-source evidence map and are used here only to compare and interpret its findings.

### 4.2. Neonatal Transport as a Sepsis-Relevant Care Interval

The traditional view of neonatal transport as a bridge between hospitals underestimates the clinical importance of the interval itself. For a neonate with suspected infection, the transport period may coincide with early physiological deterioration, diagnostic uncertainty, vascular access decisions, culture collection, antimicrobial administration, respiratory escalation, perfusion assessment, glucose correction, thermal instability, and preparation for admission to a higher-level NICU. In this sense, transport is not simply the movement of a patient; it is a time-sensitive care interval in which early treatment may be continued, delayed, modified, or initiated.

This interpretation is biologically plausible. Neonates, particularly those born preterm or with low birth weight, have limited immune reserve, immature inflammatory regulation, fragile skin and mucosal barriers, reduced cardiovascular compensation, and high dependence on external respiratory and thermal support. Sepsis can amplify these vulnerabilities through endothelial dysfunction, capillary leak, myocardial depression, pulmonary instability, microcirculatory impairment, metabolic stress, and impaired thermoregulation. During transport, these mechanisms may become visible as increasing oxygen requirement, apnea, hypotension, poor perfusion, hypoglycaemia, acidosis, hypothermia, or need for urgent escalation.

The review therefore supports a pathogenesis-informed interpretation of neonatal transport. Transport-phase variables should not be viewed as isolated operational metrics. Respiratory instability, perfusion abnormalities, temperature drift, glucose derangement, adverse events, and environmental stress may represent different expressions of a shared vulnerability in a critically ill neonate. When infection is suspected, these variables should be interpreted together and communicated clearly across the care pathway.

This biological and operational rationale should not be confused with evidence of effect. Mixed transport populations, diverse indications, and the absence of sepsis-defined exposures and outcomes mean that the available literature cannot establish whether neonatal transport independently modifies sepsis outcomes. The hypothesis requires direct prospective testing. Accordingly, conceptual relevance to sepsis, relatively direct evidence about transport processes, and direct evidence of an effect on sepsis-specific outcomes are treated as three separate constructs; only the third would support a sepsis-specific effect inference, and no such evidence was identified.

### 4.3. Proposed Pathogenesis-Informed Transport-Sepsis Framework

**Figure 2:**
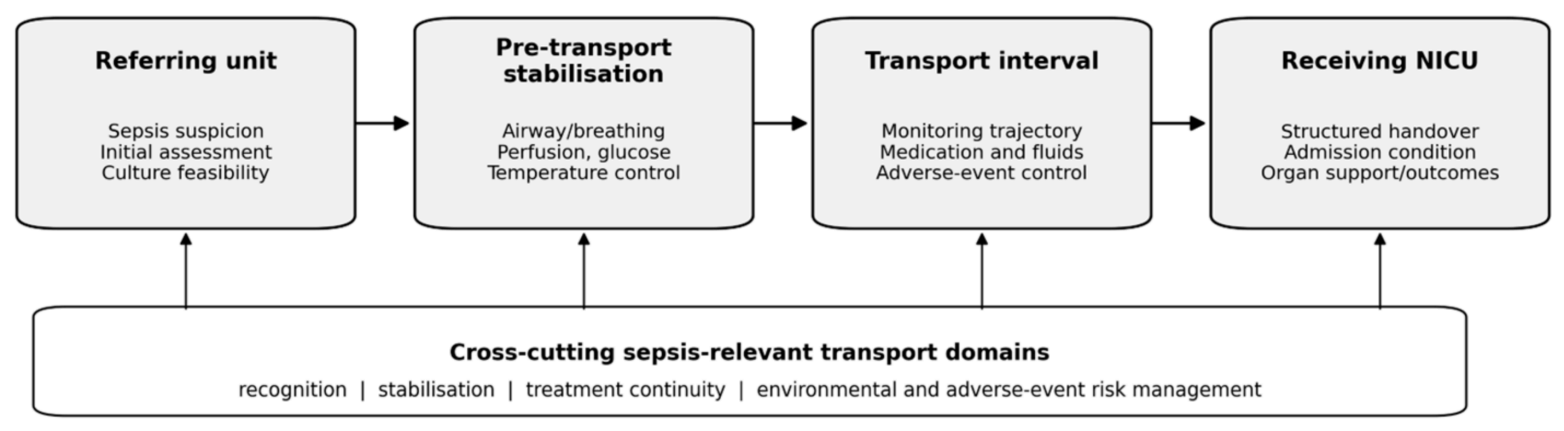
Conceptual framework for interhospital neonatal transport as a potentially sepsis-relevant care interval, from the referring unit to the receiving neonatal intensive care unit (NICU).

As an author-derived, hypothesis-generating interpretation of the synthesis, neonatal transport can be conceptualised as a pathogenesis-informed care interval organised around four linked components: recognition, stabilisation, treatment continuity, and risk management. The proposed structure is summarised in Figure 2.

The first component is recognition. Transport teams may be among the first specialist clinicians to reassess a neonate whose clinical condition is evolving. In suspected sepsis, recognition requires repeated evaluation of respiratory status, perfusion, temperature, glucose, maternal and perinatal infection risk factors, and laboratory information, when available, along with the clinical trajectory before retrieval. Documentation of the time of first sepsis concern is essential, because it provides the anchor point for evaluating treatment timing.

The second component is stabilisation. Airway, breathing, circulation, temperature, glucose, vascular access, and procedural needs should be assessed before departure and during transfer. Included sources show that respiratory support, temperature-targeted treatment, transport scoring, and adverse-event surveillance can be delivered or measured during transport [19,20,26,30,35,42,46]. This supports feasibility of measurement, not a sepsis-specific benefit. Adjacent literature—including a systematic review of urgent neonatal intubation and a protocol for an active-cooling trial—shows that procedural and thermal interventions continue to be evaluated outside the included evidence map [57,58].

The third component is treatment continuity. Culture status, antimicrobial timing, medication administration, and reasons for delay should be recorded and transmitted. This is one of the weakest areas in the current evidence base. Without these variables, it is impossible to determine whether transport improved continuity of sepsis care or contributed to avoidable delay. Future datasets should therefore include antibiotic name, dose, route, administration time, culture status, and timing relative to first clinical suspicion.

The fourth component is risk management. This includes adverse-event prevention, environmental exposure reduction, equipment readiness, transport-mode selection, communication, structured handover, and family-centred care. Environmental variables such as noise, vibration, thermal fluctuation, and transport duration should be linked with physiological trajectories rather than treated as purely technical measurements. Similarly, handover should be assessed not only as a communication act but as a mechanism for preserving diagnostic and therapeutic continuity.

This framework is a research and documentation proposal, not a clinical guideline or validated sepsis bundle. Each component requires evaluation in infection-defined cohorts before practice effects can be inferred.

### 4.4. Implications for Clinical Practice and Quality Improvement

The findings suggest candidate priorities for prospective research and locally governed quality-improvement measurement rather than validated practice requirements. Within those contexts, transport services may consider sepsis suspicion as a structured communication category. When infection is possible, referral and handover should explicitly include maternal risk factors, neonatal signs of deterioration, time of first concern, cultures obtained or pending, antibiotic administration, respiratory trajectory, perfusion status, temperature, glucose, vascular access, and expected escalation needs.

Second, admission condition should be interpreted as an outcome of the entire pre-transport and transport pathway, not merely as a baseline characteristic at arrival. Scores and physiological markers such as temperature, oxygenation, perfusion, glucose, blood pressure, and respiratory support can help characterise whether the infant arrived stabilised, unchanged, or deteriorated. This is particularly relevant in suspected sepsis, where clinical decline may be rapid and multifactorial.

Third, adverse events should be documented with timing and context. A simple list of events is insufficient. Transport systems should distinguish pre-existing instability, transport-emergent deterioration, procedural complications, equipment problems, communication failures, and treatment delays. In suspected sepsis, this distinction is essential because deterioration may reflect disease progression, incomplete stabilisation, environmental stress, or preventable system failure.

Fourth, environmental exposure should be recognised as a quality-improvement domain. Current evidence does not show that reducing sound, vibration, or thermal fluctuation improves sepsis outcomes, but these exposures are measurable and potentially modifiable. Transport services can begin by identifying high-exposure contexts, improving equipment securing, optimising incubator performance, minimising unnecessary handling, and integrating exposure monitoring into research protocols. Contextual technical and intrahospital-transport studies have also explored incubator thermoregulation and hypothermia prediction, but their methods or settings fall outside the eligibility definition [59,60].

Finally, family-centred communication should be embedded in transport quality. Parents should receive clear information about the reason for transfer, the suspected clinical problem, stabilisation performed, destination, communication pathway, and expected next steps. Although family-centred care is not a sepsis treatment, it is ethically important and may improve continuity, trust, and shared understanding during a highly stressful clinical event.

### 4.5. Implications for Future Research

The most important research implication is the need for prospective multicentre studies using harmonised transport-sepsis variables. Future studies should not merely ask whether neonatal transport is safe in general. They should test whether specific transport-phase processes influence clinically meaningful outcomes in neonates with suspected or confirmed infection.

A proposed, hypothesis-generating transport–sepsis dataset for prospective evaluation could include time of first sepsis concern, maternal and perinatal infection risks, clinical signs prompting suspicion, culture status, antimicrobial name and timing, reasons for antimicrobial delay, respiratory support, oxygenation trajectory, perfusion assessment, blood pressure, capillary refill, glucose values, temperature trajectory, fluid boluses, vasoactive support, adverse events, environmental exposure when measurable, team type, transport mode, transport duration, structured handover, admission condition, organ support, length of stay, and mortality. This proposal has not been validated and should not be treated as an evidence-based minimum standard.

Future studies should also distinguish direct from indirect sepsis evidence. Direct studies should enrol neonates with suspected or confirmed sepsis, report antimicrobial and culture timelines, and evaluate outcomes such as shock progression, ventilation, vasoactive support, mortality, and length of stay. Indirect studies should continue to examine transport stabilisation, environmental stress, adverse events, and communication quality, but should incorporate infection-related variables whenever possible.

The role of telemedicine deserves specific evaluation. Remote neonatal specialist input may improve triage, early recognition, stabilisation priorities, culture and antimicrobial decisions, and preparation of the receiving NICU. However, current evidence remains insufficient to determine whether telemedicine changes transport outcomes in sepsis-relevant neonatal cohorts.

Environmental research should move beyond exposure measurement alone. Future studies should link noise, vibration, light, thermal variation, transport mode, and duration with physiological instability, respiratory escalation, perfusion changes, glucose abnormalities, adverse events, admission condition, and outcome. Only then can environmental risk management become clinically interpretable rather than merely descriptive.

Finally, research should be adapted to resource-limited settings. Many neonatal deaths occur where specialised teams, incubators, monitoring, and rapid referral systems may be unavailable. In these settings, low-cost interventions such as thermal protection, structured referral, glucose assessment, basic perfusion evaluation, oxygenation monitoring, and timely antimicrobial coordination may be especially important. Future work should evaluate pragmatic transport-sepsis pathways that are feasible across different health-system contexts. A contextual systematic review of low-cost transport interventions in sub-Saharan Africa supports the need for resource-sensitive evaluation, whereas case-based reports illustrate feasibility in selected air and pre-hospital scenarios without supporting generalisable effectiveness conclusions [61,62,63,64].

### 4.6. Strengths and Limitations

This scoping review addresses an underdeveloped intersection between neonatal transport and early sepsis care, reports the retained multi-source search strategies, applies explicit eligibility criteria, independently screens records with two reviewers, and separates the 39 included sources from contextual literature. It also brings together clinical, organisational, environmental, and communication domains within one framework.

The reviewer-derived evidence-relationship classification and retained source-level summary make explicit which transport variables were reported by included sources, which gaps were identified through synthesis, and which elements are proposed as hypothesis-generating research priorities. The classification does not assess methodological quality or certainty. Reframing the study as a scoping review avoids implying an effectiveness synthesis that the underlying literature cannot support.

The limitations are substantial. Most included sources did not enrol suspected or confirmed sepsis populations, so clinical implications are indirect. The 2020 start date prioritised contemporary systems but excludes earlier transport evidence; English-language and peer-reviewed publication restrictions may introduce language and publication bias. Grey literature, including theses, was not searched.

The protocol was not prospectively registered, and preliminary exploratory mapping preceded finalisation of the working protocol and charting matrix. Although no changes are documented after that finalisation point, the retained record cannot reconstruct whether wording or operational rules evolved during the preliminary stage. Although two reviewers independently selected sources, source-level charting was undertaken by one author rather than in duplicate. Query-level retrieval counts, database exports, and source-specific identification logs were not retained, which limits independent reconstruction of the search yield. These features increase the possibility of selection, transcription, and interpretive error.

No formal critical appraisal was incorporated into source selection, weighting, or synthesis. The evidence-relationship categories therefore indicate proximity to the sepsis question and not methodological quality or certainty. GRADE was not applied because the revised purpose is evidence mapping and no comparable outcome-specific body of effect estimates was identified. The review therefore describes the extent and character of reporting but does not provide certainty estimates for intervention effects.

Publication bias could not be evaluated statistically. Positive or technically feasible transport experiences may be more likely to appear in the published literature, and selective reporting may favour available operational outcomes over absent culture, antimicrobial, or infection-defined outcomes. Database indexing and variable terminology may also have led to missed sources.

Finally, countries, service models, team composition, equipment, distance, and resource availability varied markedly. Technical exposure measurements were useful for mapping the transport environment but cannot be interpreted as evidence of clinical or sepsis-specific effect.

### 4.7. Overall Interpretation

The contemporary literature supports viewing interhospital neonatal transport as a measurable clinical and organisational interval that may intersect with early sepsis care. It does not establish that transport itself, or any specific transport strategy, improves neonatal sepsis outcomes.

The immediate value of this scoping review is to distinguish differing degrees of evidentiary proximity to neonatal sepsis and to define candidate variables for future prospective, infection-defined studies. Any practice recommendations should remain consistent with established neonatal transport and sepsis guidance rather than be derived from this evidence map alone.

## 5. Conclusions

This scoping review examined 39 contemporary sources on interhospital neonatal transport. The literature describes active clinical care, service organisation, respiratory and thermal support, medication and fluids, adverse events, environmental exposure, and communication, but is predominantly indirect with respect to neonatal sepsis.

Culture status, antimicrobial timing, repeated perfusion and glucose measures, and structured infection-specific handover were rarely reported. No included source established that a transport intervention independently improved a sepsis-specific outcome.

The proposed transport–sepsis framework and dataset should therefore be understood as unvalidated, hypothesis-generating research and documentation proposals, not as a care bundle, an evidence-based minimum standard, or proof of benefit.

Prospective multicentre studies should enrol infection-defined populations, use harmonised transport-phase variables, and link them to admission condition, organ support, length of stay, and mortality before clinical effectiveness claims are made.

## Figures and Tables

**Table 1:** Characteristics and relationship to sepsis evidence of the 39 included sources.

Study [Reference]	Country/Setting	Design	Transport Domain	Relationship to Sepsis Evidence	Reviewer-Derived Primary Contribution to Evidence Map
Marsinyach Ros et al., 2020 [11]	Spain/specialised neonatal transport programme	Service evaluation	Quality metrics	Indirect organisational	Reports predefined quality indicators for neonatal transport performance.
Chakkarapani et al., 2020 [12]	Canada/interfacility neonatal transport	Observational study	Procedures and stabilisation time	Indirect clinical	Reports that procedural interventions and stabilisation time are measurable components of pre-transport and transport-phase care.
Curfman et al., 2020 [13]	United States/paediatric and neonatal transport	Implementation study	Telemedicine	Indirect organisational	Reports telemedicine as a tool for remote assessment, communication, and decision support during neonatal transport.
Leemann et al., 2020 [14]	Switzerland/neonatal transport service	Retrospective cohort	System organisation	Indirect organisational	Provides European data on transport activity, organisation, and service characteristics.
Tette et al., 2020 [15]	Ghana/outborn neonates	Cross-sectional study	Low-resource transport and outcomes	Direct transport-process/indirect sepsis-outcome	Reports associations between transport conditions and admission status with neonatal outcomes in a resource-limited setting.
Falsaperla et al., 2021 [16]	Italy/neonatal emergency transport	Observational study	Emergency transport outcomes	Indirect clinical	Evaluates the impact of neonatal emergency transport organisation on morbidity and mortality indicators.
Singh et al., 2021 [17]	India/tertiary referral centre	Prospective observational study	Transport characteristics and mortality	Direct transport-process/indirect sepsis-outcome	Identifies transport and admission-related predictors of mortality among referred neonates.
Partridge et al., 2021 [18]	United Kingdom/ambulance transport	Technical observational study	Noise and vibration monitoring	Indirect technical	Reports feasibility of measuring transport-related environmental exposure.
Belli et al., 2022 [19]	Italy/neonatal transport	Observational study	HFOV during transport	Indirect clinical	Reports feasibility of advanced respiratory support during neonatal transport.
Cavallin et al., 2022 [20]	Low-resource setting/ambulance-transferred neonates	Retrospective observational study	TOPS and outcome prediction	Indirect clinical	Reports associations between temperature, oxygenation, perfusion, and glucose status with post-transport mortality risk.
Greene et al., 2022 [21]	Regional care network	Observational study	Vital-sign trajectories	Indirect clinical	Reports that physiological trajectories during transport can be characterised and related to risk.
Mishra et al., 2022 [22]	India/tertiary paediatric emergency	Observational study	Referral quality and admission status	Indirect clinical	Reports associations among referral quality, admission condition, and neonatal outcomes.
Qu et al., 2022 [23]	China/outborn full-term infants	Retrospective cohort	Transport scoring systems	Indirect clinical	Compares transport scores for predicting mortality after neonatal transfer.
Rasania et al., 2022 [24]	India/tertiary care hospital	Observational study	Transport facilities and outcomes	Indirect clinical	Contributes evidence on transport facilities and neonatal outcomes in a tertiary-care setting.
Dockery and Harrison, 2024 [25]	Regional transport service	Service evaluation	Ventilation trends	Indirect clinical	Describes changes in neonatal ventilation practices within a transport service.
Garnaud et al., 2024 [26]	France/neonatal transport	Before-and-after QI study	Servo-controlled hypothermia	Indirect clinical	Reports how time-sensitive treatment can be protocolised during neonatal transport.
Gardiner et al., 2024 [27]	Australia/long-distance transport	Comparative observational study	Unintended events and team type	Indirect clinical	Compares adverse events between specialised and non-specialised transport teams.
Lengauer and Grass, 2024 [28]	Single-centre neonatal transport setting	Cross-sectional study	Parent needs	Indirect experiential	Highlights family-centred communication needs during neonatal transfer.
McKissic et al., 2024 [29]	Regional paediatric critical care transport team	Descriptive study	Medications and intravenous fluids	Direct transport-process/indirect sepsis-outcome	Provides transport-phase medication and fluid data relevant to antimicrobial and stabilisation pathways.
Rana et al., 2024 [30]	Neonates with HIE	Comparative observational study	Servo-controlled vs passive cooling	Indirect clinical	Reports active temperature management as a model for protocolised transport-phase care.
Schumacher et al., 2024 [31]	Neonatal emergency transport service	Observational study	Emergency transport challenges	Indirect clinical	Describes clinical and operational complexity in neonatal emergency transports.
Solvik-Olsen et al., 2024 [32]	Helicopter incubator transport	Experimental/manikin study	Sound and vibration exposure	Indirect technical	Reports that neonates may be exposed to high sound and vibration levels during helicopter transport.
Tamez et al., 2024 [33]	Sweden/regional cohort	Cohort study	Therapeutic hypothermia, transport and outcomes	Indirect clinical	Reports associations between regionalised hypothermia care, transport, and neonatal outcomes.
Bellini et al., 2025 [34]	Italy/national survey	Survey	Transport organisation and activity	Indirect clinical	Provides national data on neonatal emergency transport organisation and activity.
Balog et al., 2025 [35]	Neonatal transport service	Observational study	HFOV with/without volume guarantee	Indirect clinical	Evaluates advanced ventilation during transport in critically ill neonates.
Huang et al., 2025 [36]	China/single centre	Observational study	Servo-controlled cooling	Indirect clinical	Reports feasibility of active cooling during transport.
Jenkinson et al., 2025 [37]	UK and Ireland	Survey	Respiratory support strategies	Indirect organisational	Reports heterogeneity in respiratory support practices during neonatal transport.
Mistry et al., 2026 [38]	Regional transport network	Implementation study	Active therapeutic hypothermia	Indirect clinical	Reports implementation of active transport-phase treatment across a regional network.
Morel et al., 2025 [39]	Near-term and term infants on CPAP	Observational study	Intervention prediction during transfer	Indirect clinical	Identifies clinical interventions and predictors of escalation during transfer.
Partridge et al., 2025 [40]	Neonatal ambulance transport	Observational/technical study	Noise and vibration exposure	Indirect technical	Quantifies environmental exposure during neonatal ambulance transport.
Perez et al., 2025 [41]	Italy/national survey	Survey	Transport ventilators	Indirect organisational	Describes ventilators used in emergency neonatal transport and equipment heterogeneity.
Risso et al., 2025 [42]	Italy/emergency transport	Observational study	Adverse events	Indirect clinical	Evaluates the impact of adverse events on neonatal emergency transport.
Schwarz et al., 2025 [43]	Switzerland/neonatal transport	Retrospective cohort	Clinical outcomes and risk factors	Indirect clinical	Reports transport-associated outcomes and risk factors in a neonatal cohort.
Sohane et al., 2025 [44]	Neonatal transfer service	Quality-improvement study	Team mobilisation	Indirect organisational	Reports that transport team mobilisation time can be improved through QI methods.
Trulsen et al., 2025 [45]	Arctic Norway/population-based cohort	Population-based study	Air transport	Indirect organisational	Provides long-distance air-transport data in a geographically challenging neonatal network.
Lourens et al., 2025 [46]	South Africa/aeromedical transfers	Retrospective review	Respiratory support during air transfer	Indirect clinical	Describes respiratory support and interventions during aeromedical neonatal and infant transfers.
Butler et al., 2025 [47]	Preterm neonates	Exploratory analysis	Transport-related IVH risk	Indirect clinical	Reports that transport exposure may have measurable physiological consequences.
Lichtsinn et al., 2026 [48]	Neonates with CDH	Observational study	Interhospital transport characteristics	Indirect clinical	Describes transport features in a high-risk physiological subgroup.
Gibb et al., 2026 [49]	Fixed-wing neonatal transport	Observational/technical study	Sound and vibration exposure	Indirect technical	Quantifies sound and vibration exposure during fixed-wing neonatal transport.

Abbreviations: CDH, congenital diaphragmatic hernia; CPAP, continuous positive airway pressure; HFOV, high-frequency oscillatory ventilation; HIE, hypoxic–ischaemic encephalopathy; IVH, intraventricular haemorrhage; QI, quality improvement; TOPS, Temperature, Oxygenation, Perfusion, and Sugar.

**Table 2:** Evidence map of contemporary transport-phase domains potentially relevant to early neonatal sepsis care.

Domain	Transport Variables Represented in Included Sources	Relationship to Sepsis Evidence	Extent and Character of Evidence	Key Evidence Gap
System organisation and transport-team performance	Team type; mobilisation time; referral pathway; benchmarking	Indirect; organisational proximity	Several observational/service/survey/QI sources	Operational indicators rarely linked to infection-defined outcomes
Medication, fluids, and infection-related documentation	Medication and intravenous-fluid records; diagnoses and treatment at referral	Closest conceptual proximity; sepsis-outcome evidence indirect	One medication/fluids source; sparse infection reporting	Culture and antimicrobial timelines are not standardised
Respiratory and procedural stabilisation	CPAP; ventilation; HFOV; airway interventions; escalation	Indirect clinical	Multiple clinical and survey sources	Infection-defined respiratory subgroups are rarely reported
Haemodynamic, metabolic, and thermal stabilisation	Perfusion; blood pressure; glucose; temperature; transport scores	Indirect clinical	Multiple score/temperature sources; sparse perfusion/glucose data	Repeated trajectories are incompletely captured
Adverse events and physiological deterioration	Desaturation; airway/access problems; equipment events; escalation	Indirect clinical	Several heterogeneous observational sources	Event timing and relation to disease progression are inconsistent
Environmental exposures	Noise; vibration; thermal drift; duration; air/ground constraints	Indirect technical	Several exposure-monitoring sources	Clinical thresholds and consequences remain uncertain
Communication, handover, telemedicine, and family experience	Referral information; infection suspicion; structured handover	Indirect, non-clinical	Few service, handover, or parent sources	No harmonised infection-specific handover dataset
Post-transport outcomes	Admission condition; organ support; length of stay; mortality	Indirect unless infection-defined	Several heterogeneous cohorts	Outcomes are seldom linked to transport-phase infection timelines

Abbreviations: CPAP, continuous positive airway pressure; HFOV, high-frequency oscillatory ventilation; QI, quality improvement.

## Data Availability

The retained source-classification and summary data for this scoping review are provided in Supplementary File S3. Query-level search exports, source-specific retrieval counts, and Google Scholar ranking cut-offs were not retained and are not available.

## Supplementary Materials

The following supporting information can be downloaded at https://www.mdpi.com/article/10.3390/medicina62091753/s1, Supplementary File S1: PRISMA-ScR checklist [8]; Supplementary File S2: retained search strategies, eligibility clarifications, and documentation limits; Supplementary File S3: source-classification and summary matrix, comprising the included sources [11,12,13,14,15,16,17,18,19,20,21,22,23,24,25,26,27,28,29,30,31,32,33,34,35,36,37,38,39,40,41,42,43,44,45,46,47,48,49] and the contextual or methodological sources [1,2,3,4,5,6,7,8,9,10,50,51,52,53,54,55,56,57,58,59,60,61,62,63,64].

## Abbreviations

The following abbreviations are used in this manuscript:

CDH	Congenital diaphragmatic hernia
CPAP	Continuous positive airway pressure
HFOV	High-frequency oscillatory ventilation
HIE	Hypoxic–ischaemic encephalopathy
IVH	Intraventricular haemorrhage
NICU	Neonatal intensive care unit
PCC	Population–Concept–Context
PRISMA-ScR	Preferred Reporting Items for Systematic Reviews and Meta-Analyses extension for Scoping Reviews
QI	Quality improvement
TOPS	Temperature, Oxygenation, Perfusion, and Sugar
