# Supplementary material for: Contemporary Evidence on Interhospital Neonatal Transport as a Potentially Sepsis-Relevant Care Interval: A Scoping Review
Source: Medicina (Kaunas). 2026 Sep 11;62(9):1753. doi: 10.3390/medicina62091753 (PMC13609382; doi:10.3390/medicina62091753)
Supplement: Supplementary file 1 [file medicina-62-01753-s001.zip › Supplementary_S1-S2_Round2_Tracked.pdf]

Contemporary Evidence on Interhospital Neonatal Transport as a Potentially Sepsis-Relevant Care Interval: A Scoping Review

Contents:

S1 PRISMA-ScR checklist; S2 retained search strategies, eligibility, review-process details, and documentation limits

Companion file: S3 source-classification and summary matrix (.xlsx)

S1. PRISMA-ScR Checklist

Reporting guideline: Tricco AC et al. PRISMA Extension for Scoping Reviews (PRISMA-ScR): Checklist and Explanation. Ann Intern Med. 2018;169:467–473. doi:10.7326/M18-0850.

Line-number basis: continuous line numbering in the clean revised manuscript. Word may repaginate slightly across software versions, but the numbered section locations remain identifiable.

Section / topic	Item	PRISMA-ScR requirement	Location in revised manuscript
**TITLE**	1	Identify the report as a scoping review.	Title, lines 1–3
**ABSTRACT**	2	Provide a structured summary including background, objectives, eligibility, sources, charting, results, and conclusions.	Abstract, lines 31–53
**INTRODUCTION—Rationale**	3	Describe the rationale in the context of what is already known and explain why a scoping approach is appropriate.	Introduction, lines 57–79
**INTRODUCTION—Objectives**	4	State the questions and objectives with reference to population, concept, context, or other relevant elements.	Introduction, lines 80–83; Methods, lines 101–115
**METHODS—Protocol and registration**	5	Indicate whether a protocol exists, where it can be accessed, and registration information if available.	Methods, lines 97–100; no prospective registration
**METHODS—Eligibility criteria**	6	Specify source characteristics and eligibility criteria, with a rationale.	Methods, lines 116–138
**METHODS—Information sources**	7	Describe all information sources and the date of the most recent search.	Methods, lines 139–154; last search 30 June 2026
**METHODS—Search**	8	Present the full electronic search strategy for at least one database, including limits.	Methods, lines 155–201; full PubMed strategy and retained source-specific strings in Supplementary File S2; query-level yields unavailable
**METHODS—Selection of sources**	9	State the process for selecting sources of evidence.	Methods, lines 202–214
**METHODS—Data charting process**	10	Describe the charting method and how data were confirmed.	Methods, lines 215–226; single-author source-level charting
**METHODS—Data items**	11	List and define the variables sought and assumptions or simplifications.	Methods, lines 215–238; retained fields in Supplementary File S3
**METHODS—Critical appraisal**	12	If done, describe the rationale, methods, and use of critical appraisal.	Methods, Section 2.8; no formal critical appraisal incorporated into selection, weighting, or synthesis
**METHODS—Synthesis**	13	Describe methods for handling and summarising charted data.	Methods, lines 227–242
**RESULTS—Selection**	14	Give numbers screened, assessed, and included, with reasons for exclusions.	Results, lines 269–282; Figure 1; aggregate identification counts
**RESULTS—Characteristics**	15	Present characteristics of each source and provide citations.	Results, lines 283–295; Table 1
**RESULTS—Critical appraisal**	16	If done, present appraisal results.	Not applicable; no formal critical appraisal was incorporated into the review synthesis
**RESULTS—Individual sources**	17	Present relevant charted data for each source.	Table 1; Supplementary File S3 source-classification and summary matrix
**RESULTS—Synthesis**	18	Summarise and/or present charted results as they relate to the questions and objectives.	Results, lines 296–392; Tables

			1–2
**DISCUSSION—Summary**	19	Summarise the main results and relate them to the questions and objectives.	Discussion, lines 394–412
**DISCUSSION—Limitations**	20	Discuss limitations of the scoping-review process.	Discussion, lines 559–596
**DISCUSSION—Conclusions**	21	Provide a general interpretation and implications or next steps.	Discussion and Conclusions, lines 597–620
**FUNDING**	22	Describe funding sources and the role of funders.	Funding statement, lines 631–634; the APC funder had no role in the review; funding of included sources was not charted

S2. Search, Eligibility, and Review-Process Details

S2.1. Scope and search date

The review covers contemporary evidence published from 1 January 2020 to 30 June 2026.The final search date was 30 June 2026. The period was chosen to focus on current transport equipment, telemedicine, advanced ventilation, environmental monitoring, and structured service models. It should not be interpreted as a historical review of neonatal transport, and omission of earlier evidence is acknowledged as a limitation.

S2.2. Information sources

The bibliographic databases were PubMed/MEDLINE, Embase, Scopus, Web of Science, and the Cochrane Library. Google Scholar was used as a supplementary academic search engine. Backward citation searching followed database screening and full-text identification. Reviews were considered relevant for citation chasing when they addressed neonatal interhospital transport, neonatal retrieval systems, transport safety, transport variables, or transport-phase clinical and organisational processes.The retained project files did not preserve query-level retrieval counts, database exports, source-specific identification counts, or Google Scholar ranking cut-offs; the selection flow is therefore reported using the reconciled aggregate record. Re-running the searches after the original search date would interrogate updated databases and could not reproduce the historical source-specific yields.

S2.3. Full search strategies

The PubMed/MEDLINE strategy was

(("Infant, Newborn"[Mesh] OR neonat* OR newborn* OR preterm OR premature)

AND ("Transportation of Patients"[Mesh] OR "Patient Transfer"[Mesh] OR transport* OR transfer* OR retrieval OR "mobile intensive care" OR ambulance OR aeromedical OR interhospital) AND (sepsis OR infection OR "suspected sepsis" OR antibiotic* OR antimicrobial* OR culture* OR stabilization OR stabilisation OR respiratory OR ventilation OR hemodynamic OR haemodynamic OR perfusion OR glucose OR hypothermia OR temperature OR "adverse event*" OR handover OR communication OR noise OR vibration OR environmental)) AND ("2020/01/01"[Date - Publication] : "2026/06/30"[Date - Publication])

For Embase, the search was adapted as

('newborn'/exp OR neonat*:ti,ab OR newborn*:ti,ab OR premature:ti,ab OR preterm:ti,ab) AND ('patient transport'/exp OR transport*:ti,ab OR retrieval:ti,ab OR interhospital:ti,ab OR ambulance:ti,ab OR aeromedical:ti,ab) AND (sepsis:ti,ab OR infection:ti,ab OR antibiotic*:ti,ab OR antimicrobial*:ti,ab OR culture*:ti,ab OR respiratory:ti,ab OR ventilation:ti,ab OR hemodynamic:ti,ab OR haemodynamic:ti,ab OR perfusion:ti,ab OR glucose:ti,ab OR temperature:ti,ab OR hypothermia:ti,ab OR "adverse event*":ti,ab OR vibration:ti,ab OR noise:ti,ab OR handover:ti,ab OR communication:ti,ab) AND [2020-2026]/py

For Scopus, the following title–abstract–keyword strategy was used

TITLE-ABS-KEY (neonat* OR newborn* OR preterm OR premature) AND TITLE-ABS-KEY(transport* OR transfer* OR retrieval OR interhospital OR ambulance OR aeromedical OR "mobile intensive care") AND TITLE-ABS-KEY(sepsis OR infection OR antibiotic* OR antimicrobial* OR culture* OR respiratory OR ventilation OR hemodynamic OR haemodynamic OR perfusion OR glucose OR hypothermia OR temperature OR "adverse event*" OR vibration OR noise OR communication OR handover) AND PUBYEAR > 2019 AND PUBYEAR < 2027

For Web of Science, the topic search was

TS=(neonat* OR newborn* OR preterm OR premature) AND TS=(transport* OR transfer* OR retrieval OR interhospital OR ambulance OR aeromedical OR "mobile intensive care") AND TS=(sepsis OR infection OR antibiotic* OR antimicrobial* OR culture* OR respiratory OR ventilation OR hemodynamic OR haemodynamic OR perfusion OR glucose OR temperature OR hypothermia OR "adverse event*" OR vibration OR noise OR communication OR handover)

Timespan: 2020–2026

For the Cochrane Library, the following search combinations were used:

“neonatal transport”, “neonatal retrieval”, “interhospital neonatal transfer”, “neonatal transport AND sepsis”, “neonatal transport AND respiratory support”, “neonatal transport AND hypothermia”, and “neonatal transport AND adverse events”.

Google Scholar was used as a supplementary discovery source with targeted combinations of the following terms: “neonatal transport sepsis”, “interhospital neonatal transport”, “neonatal retrieval”, “neonatal transport respiratory support”, “neonatal transport adverse events”, “neonatal transport vibration”, “neonatal transport noise”, “neonatal transport handover”, “neonatal transport thermoregulation”, “neonatal transport antibiotics”, and “neonatal transport telemedicine”. Query-level result counts and ranking cut-offs were not retained; no source-specific yield has been reconstructed retrospectively**.**

S2.4. Eligibility clarifications

Criterion	Final specification
**Population**	Neonates/newborns or a separately extractable neonatal subgroup.
**Context**	Interhospital transport, neonatal retrieval, or mobile neonatal intensive care by ground or air; exclude intrahospital transport.
**Language and publication status**	English-language, peer-reviewed full-text reports.
**Eligible designs**	Clinical observational studies, registries, surveys, service evaluations, quality-improvement and implementation studies, and technical/experimental studies directly measuring the neonatal transport environment.
**Excluded source types**	Doctoral theses and other grey literature; narrative and systematic reviews/meta-analyses; guidelines; protocols; and individual case reports.
**Contextual sources**	Excluded source types could inform the Introduction, Discussion, methods, or citation chasing, but did not contribute observations to Results and were not counted among the 39 included sources.

S2.5. Selection, charting, and interpretive safeguards

- Two reviewers independently screened titles/abstracts and full texts. Reviewers resolved disagreements through discussion and consensus.
- Backwards-citation records were deduplicated and assessed using the same eligibility criteria.
- A third author charted source-level data using the predefined matrix. Charting was not duplicated and this is reported as a limitation. Retained fields were limited to citation, year, country or setting, design or source type, primary transport domain, relationship to sepsis evidence, reviewer-derived primary contribution to the evidence map, review decision, and source or DOI URL. A completed source-level extraction of sample sizes, outcome definitions, and study-specific limitations was not retained and was not reconstructed retrospectively.
- Only the 39 included sources contribute observations in Results. Contextual literature is confined to the Introduction and Discussion.
- Relationship-to-sepsis labels describe evidentiary proximity, not methodological quality or certainty. Direct transport-process/indirect sepsis-outcome evidence denotes transport processes, infection-relevant diagnoses, treatment processes, or transport conditions reported with clinical outcomes that were not sepsis-specific. Indirect clinical evidence denotes transport-phase clinical variables in mixed neonatal populations without an explicit sepsis-specific process–outcome link; indirect organisational evidence denotes service configuration, workflow, performance, or implementation; indirect experiential evidence denotes parent or family perspectives; and indirect technical evidence denotes environmental or equipment exposures without a clinical sepsis outcome. Each source was assigned one primary evidence category according to the contribution most closely aligned with the review question.
- The eight transport domains were initially derived deductively from the PCC concept and predefined charting fields and then refined descriptively during synthesis. Each source was assigned one primary transport domain according to its dominant transport-phase focus; secondary contributions could inform the narrative synthesis but were not multiply counted. No formal critical appraisal was incorporated into source selection, weighting, or synthesis, and no low/moderate/high or GRADE certainty ratings were assigned.

S2.6. Documentation limits

- The retained search documentation supports the reported database-specific strings, supplementary discovery terms, date limits, and reconciled aggregate PRISMA-ScR counts. It does not support reconstruction of query-level retrieval counts, original database exports, source-specific identification yields, or Google Scholar ranking cut-offs. These elements were therefore disclosed as unavailable rather than inferred retrospectively. Supplementary File S3 is a source-classification and summary matrix, not a participant-level outcome extraction. Its primary-contribution column is a reviewer-derived interpretation used for this evidence map rather than a verbatim statement of each source’s primary objective or finding.
